# Colon Epithelial MicroRNA Network in Fatty Liver

**DOI:** 10.1155/2018/8246103

**Published:** 2018-09-24

**Authors:** Paule V. Joseph, Sarah K. Abey, Dan Wang, Nicolaas H. Fourie, Natnael D. Kenea, Tatyana G. Vishnyakova, Jeffrey M. Robinson, Kristen R. Weaver, Christina M. Boulineaux, Hannah R. Davidson, LeeAnne B. Sherwin, Onyinyechi Ozoji, Ana F. Diallo, Paul A. Smyser, Amy P. Patterson, Wendy A. Henderson

**Affiliations:** ^1^Digestive Disorder Unit, Division of Intramural Research, National Institute of Nursing Research, National Institutes of Health, Bethesda, MD 20892, USA; ^2^Department of Laboratory Medicine, Clinical Center, National Institutes of Health, Bethesda, MD 20814, USA; ^3^National Heart Lung and Blood Institute, Bethesda, MD 20814, USA

## Abstract

**Background & Aims:**

Intestinal barrier alterations are associated with fatty liver (FL) and metabolic syndrome (MetS), but microRNA (miR) signaling pathways in MetS-FL pathogenesis remain unclear. This study investigates an epithelial-focused miR network in colorectal cell models based on the previously reported MetS-FL miR trio of hsa-miR-142-3p, hsa-miR-18b, and hsa-miR-890.

**Methods:**

Each miR mimic construct of MetS-FL miR trio was transfected into human colorectal cells, CRL-1790 or Caco-2. Global miRNome changes posttransfection were profiled (nCounter® Human v3 miRNA, NanoString Technologies). Changes in barrier (transepithelial electrical resistance, TEER) and epithelial cell junction structure (Occludin and Zona Occludens-1/ZO-1 immunofluorescence staining-confocal microscopy) were examined pre- and posttransfection in Caco-2 cell monolayers. A signaling network was constructed from the MetS-FL miR trio, MetS-FL miR-induced colorectal miRNome changes, ZO-1, and Occludin.

**Results:**

Transfection of CRL-1790 cells with each MetS-FL miR mimic led to global changes in the cellular miRNome profile, with 288 miRs being altered in expression by more than twofold. Eleven miRs with known cytoskeletal and metabolic roles were commonly altered in expression by all three miR mimics. Transfection of Caco-2 cell monolayers with each MetS-FL miR mimic induced barrier-associated TEER variations and led to structural modifications of ZO-1 and Occludin within epithelial cell junctions. Pathway analysis incorporating the MetS-FL miR trio, eleven common target miRs, ZO-1, and Occludin revealed a signaling network centered on TNF and AKT2, which highlights injury, inflammation, and hyperplasia.

**Conclusions:**

Colon-specific changes in epithelial barriers, cell junction structure, and a miRNome signaling network are described from functional studies of a MetS-FL miR trio signature.

## 1. Introduction

Metabolic Syndrome (MetS) arises from systemic metabolic perturbations characterized by dyslipidemia and central obesity [1]. MetS is associated with an increased risk of developing cardiovascular disease, liver fibrosis and cancer, colon cancer, and breast cancer [2–5]. MetS characteristics are highly prevalent among individuals with nonalcoholic fatty liver disease (NAFLD), particularly those who are obese (Body Mass Index or BMI ≥ 30) or have insulin resistance [6]. A meta-analysis of studies demonstrated that NAFLD patients had increased intestinal permeability compared to healthy controls. Intestinal inflammation and upregulation of tumor necrosis factor-*α* (TNF*α*), common in obese individuals with MetS and NAFLD, are also associated with intestinal leakage [7]. Pathophysiology of MetS and NAFLD has been attributed to changes in intestinal epithelial barriers, leading to altered intestinal permeability and translocation of microbes or microbial products, such as lipopolysaccharide (LPS) [8, 9]. Structural proteins, such as ZO-1 and Occludin, compose a cytoskeletal-supported cell junction network that maintains transport across the intestinal epithelial barrier and regulates permeability during normal physiology [10, 11]. Expression and localization of these structural proteins are known to be regulated by certain microRNAs (miRs), such as miR-142-3p, which regulates Rac1 [12]. We previously reported altered expression of miR-142-3p, miR-18b, and miR-890 in a cohort of patients with fatty liver (FL), who also met established criteria for MetS as defined by the International Diabetes Federation [1]. Also altered in this cohort were the expressions of mRNAs related to hepatocellular carcinoma progression pathways of adhesion, invasion, and metastasis [13]. Thus, these three miRs (hsa-miR-142-3p, hsa-miR-18b, and hsa-miR-890) may be indicators of epithelial barrier functional modifications in metabolically related MetS-FL.

The regulatory properties of miRs are unique in that a single miR can exert subtle effects on multiple mRNA targets simultaneously [14]. This property of miRs is increasingly recognized to be key in systemic metabolic disorders, as it allows multiple pathways to respond in concert when cells are faced with metabolic stress [15]. Furthermore, one mRNA species is susceptible to regulatory controls by several miRs; thus, a network of miRs is likely required to control a signaling pathway [16]. In MetS and NAFLD, a network of miRs is thought to act in concert across multiple organs to induce pathophysiological damage [15, 17]. Out of the three MetS-FL miRs identified in our previous cohort, the hsa-miR-142-3p has been shown to have functional multiplicity by being able to directly regulate structural proteins within the cell junction [18], participate in the initiation of LPS-induced inflammatory signaling in immune and dendritic cells [19], and be implicated in systemic metabolic stress through its upregulation in the adipose tissue of mice fed a high-fat diet [20] and in obese children [21]. Thus, a multifunctional signaling network that simultaneously affects metabolic stress and inflammation is likely involved in the regulation of genes encoding for epithelial cell junction proteins associated with altered barrier function in MetS and NAFLD.

The cytoskeletal structure that supports the intestinal epithelial cell junction consists of proteins regulated by a signaling network that responds to extracellular environments under normal physiological conditions or stress [10, 11]. In mice, a high-fat diet that induces higher levels of circulating LPS, insulin resistance, and obesity [22] also leads to altered levels of epithelial cell junction proteins, Zonula Occludens-1 (ZO-1), and Occludin, in the duodenum [23]. Altered expressions of ZO-1 and increased intestinal permeability have been observed in NAFLD patients [24]. While these observations suggest the involvement of microbial translocation and altered epithelial barriers in MetS and NAFLD, the underlying signaling network regulating these processes is not fully understood.

To facilitate the identification of an epithelial-focused functional miR network involved in altered epithelial barriers and cell junctions, we expressed each of our previously discussed MetS-FL miR trio (hsa-miR-142, hsa-miR-18b, and hsa-miR-890) in cultured epithelial cells [13, 25]. We utilized colorectal epithelial cells since our patient cohort also had evidence of altered permeability specific to the colon [26]. The human primary colorectal cell line CRL-1790 was chosen as a metabolically normal colon epithelial cell model for the following reasons: (1) CRL-1790 is a human primary, nontransformed, colorectal cell line [27], which therefore does not possess extraneously induced genetic alterations, including those related to metabolic adaptations found in immortalized cancer cell lines; (2) expression of beta-catenin (an inducer of cell migration) was shown to be very low for this cell line [28], prompting its utilization as a nonmetastatic cell line control for investigations of colorectal adenocarcinomas; (3) the cell line displays epithelial characteristics yet lacks keratin [27], a cytoskeletal protein which has been associated with many types of neoplasia [29]. Each of the miR mimics were transfected into CRL-1790 cells, and global miRNome expression profiling was performed (NanoString Technologies). In addition, we transfected each of the miR mimics into Caco-2 epithelial cell monolayers and analyzed changes in miR-induced epithelial barrier integrity by measuring transepithelial electrical resistance [30] pre- and posttransfection. Caco-2 cells have been shown to form epithelial monolayers* in vitro* resembling the intestinal barrier villi [31]. For this reason, Caco-2 cells have been found to be an excellent model for passive transcellular transport normally occurring* in vivo* [31] and to investigate intestinal barrier permeability in steatohepatitis [7]. Next, to evaluate the cytoskeletal proteins that may affect epithelial barrier function, we examined ZO-1 and Occludin structures in the epithelial cell junction and their mRNA levels post-miR mimic transfection. Finally, a signaling network was constructed from the MetS-FL miR trio, ZO-1, Occludin, and miR targets within the global miRNome affected by MetS-FL miR expressions.

## 2. Materials and Methods

### 2.1. Cell Cultures and Transfection of Cells with miR Mimics

Human colon epithelial cell lines Caco-2 and CRL-1790 were purchased from American Type Culture Collection (ATCC). Cells were cultured in Eagle's Minimum Essential Medium (EMEM; ATCC) containing 20% (Caco-2) or 10% (CRL-1790) Fetal Bovine Serum (FBS; ThermoFisher) at 37°C and 5% CO_2_. Expression of miR mimics in Caco-2 and CRL-1790 cells was carried out using DharmaFect transfection reagent (GE Dharmacon) according to the manufacturer's protocols. Mature miRNA mimic constructs (miRIDIAN™, GE Dharmacon) were dissolved in siRNA buffer (GE Dharmacon) to achieve final concentrations of 100 nM. For RNA isolation from transfected CRL-1790 cells, transfection media containing miR mimics or vehicles (siRNA buffer) were replaced with fresh feeding media at 24 hours posttransfection and cells were incubated for an additional 24 hours. Total RNAs were isolated using a microRNA isolation kit (Qiagen). The* C.elegans* cel-miR-67 mimic (GE Dharmacon) was used as a nonmammalian miR control.

### 2.2. Analysis of the Global miRNome Signaling Network

Total RNA from miR mimic-transfected cells was evaluated using nCounter® Human v3 miRNA expression assays (NanoString Technologies), as previously described (Fourie 2016). Endogenous changes of miRNAs in CRL-1790 cells expressing each of the MetS-FL miR mimics were analyzed using nSolver™ Analysis Software v3.0 (NanoString). Normalization of digital counts was performed using “Top 100” miRs with highest counts; only miRs with counts above the normalization threshold value of 43.17 (calculated from mean ±3 standard deviations of eight negative controls) were included in subsequent ratio calculations. The ratios of digital counts in cells expressing MetS-FL miR mimic to the digital counts in cells expressing control cel-miR mimic were determined. From this ratio calculation, only miRs with ratios of 2.0 or above, or -2.0 or below, were subjected to Venn analysis [33]. Venn analysis was performed with 288 miRs having these specified ratios and a diagram constructed to illustrate the number of miRs within the global cellular miRNome that were commonly targeted.

### 2.3. *In Vitro* Intestinal Permeability Assay

For each biological replicate, Caco-2 cells were seeded at 10^5^ cells per well on Millicell filters (EMD Millipore) according to manufacturer instructions. Cells were allowed to polarize into apico-basolateral monolayers for 7 days prior to transfection, and feeding media (20% FBS) was replaced every 2 days. On day 8, cells were transfected with each of the MetS-FL miR mimics, control cel-miR-67 mimic, or vehicle (siRNA buffer). TEER values were measured every day pre- and posttransfection in replicate wells using Millicell ERS-2 and electrodes (EMD Millipore). TEER values in blank wells seeded with no cells were subtracted from these TEER values and then normalized against a 24-well plate surface area per well (values were shown as Ohm-cm^2^). All TEER measurements were taken at room temperature in a tissue culture hood to maintain sterility.

### 2.4. Confocal Immunofluorescence Microscopy of Zona Occludens-1 and Occludin

Six days after transfection with miR mimics, polarized Caco-2 cells were washed with 1X phosphate-buffered saline (PBS) pH 7.4, fixed with 100% methanol, and blocked with 1% Bovine Serum Albumin (BSA) and with 5% goat serum/1% BSA in PBS. Fixed cells were permeabilized by two treatments of 0.2% Triton-X prior to staining with 2.5 *μ*g/ml rabbit anti-ZO-1 (GE Life Technologies) or 20 *μ*g/ml mouse anti-Occludin (GE Life Technologies). Subsequently, stained cells were washed in PBS and secondary antibodies were added: donkey anti-rabbit Alexa Fluor 594, donkey anti-mouse Alexa Fluor 488, control rabbit IgG, or control mouse IgG (GE Life Technologies). Images were acquired sequentially by using a 488-nm laser line and emission between 505 and 580 nm for Alexa Fluor 488, BODIPY 493/503, and BODIPY FL C12. A 594-nm laser line and emission >610 nm were used for Alexa 594. Z-series high-resolution (100 nm per pixel) images were obtained with a 63x, 1.4-numerical-aperture Plan-Apochromat oil-immersion objective under conditions avoiding bleed-through.

### 2.5. Quantitative Real-Time Polymerase Chain Reaction (qPCR)

To obtain RNA for qPCR assays, cells were seeded on 24-well plates until they reached 80% confluence. miRIDIAN miR mimics (100 nM) were added with DharmaFect 1 transfection reagent according to manufacturer protocols (GE Dharmacon). The C.* elegans* cel-miR-67 mimic was used as a control (GE Dharmacon). After 24 hours, transfection media was replaced with fresh feeding media and cells were incubated for an additional 24 hours. Total cellular RNA from at least three independent biological replicates was isolated using a miRNeasy isolation kit (Qiagen). RNA concentration was measured using Nanodrop (Thermo Scientific). For qPCR of miRs, 100 ng total RNA was used for cDNA synthesis using TaqMan® MicroRNA Reverse Transcription Kit. qPCR was performed using sequence-specific TaqMan® qPCR microRNA assay primers (Thermo Scientific) and TaqMan® Universal Master Mix. Endogenous mature miRNA expression analysis was performed in triplicate and normalized to the expression level of hsa-miR-16 as a housekeeping control. For qPCR of mRNA, 450 ng total RNA was used for cDNA synthesis by the TaqMan® Reverse Transcription Reagents using oligo d(T) and random hexamers primer mixture (Thermo Scientific). cDNA then was subjected to qPCR analysis. Endogenous mRNA expression analysis was performed in triplicate with TaqMan® Gene Expression Master Mix using Zona Occludens-1 and Occludin TaqMan® gene expression assay primers (Thermo Scientific) and normalized to the expression level of GAPDH as a housekeeping control. Samples were run on the 7900HT Real-Time PCR System (Life Technologies-Applied Biosystems) platform in the following conditions: initial activation step of 2 min at 50°C, 10 min at 95°C, followed by 40 cycles of 15 s at 95°C, and extension for 1 min at 60°C. miRNA or mRNA fold expression relative to expression in untransfected (or cel-miR mimic-transfected) cells from each biological replicate was calculated based on the ΔΔCt method [34]. Means and standard deviations were reported from three biological replicates.

## 3. Results

### 3.1. Colorectal Cellular miRNome Is Globally Affected by MetS-FL miRs

**Figure 1:**
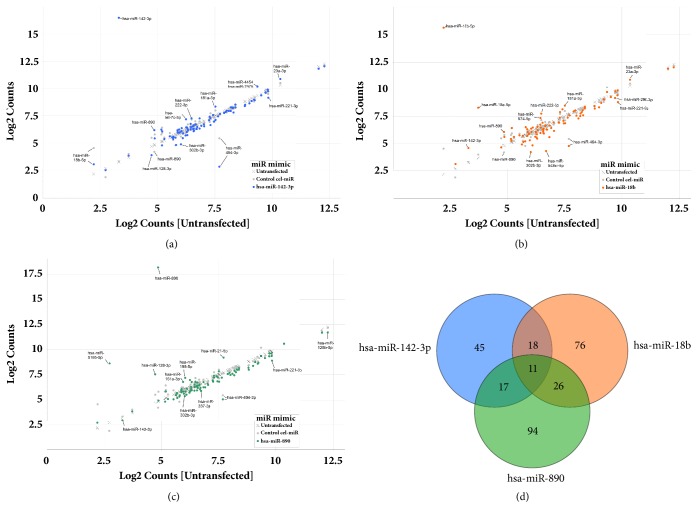
(a-c) Global alterations in colorectal cell miR levels in human epithelial CRL-1790 cells transfected with control cel-miR or MetS-FL miR mimics. (d) Common miR targets whose expression levels were altered by 2.0-fold or more following CRL-1790 cell transfection with each MetS-FL miR mimic. Eleven common miRs targeted by all three MetS-FL miRs are listed in Table 1.

In MetS and NAFLD, regulation of the intestinal barrier is essential as bacterial translocation and inflammation are thought to ensue following barrier breakage [8]. We tested whether the MetS-FL miR trio, found previously in patients with FL and altered colonic permeability, could be part of a signaling network in colon epithelial cells that form intestinal barriers. We transfected CRL-1790 cells with either the MetS-FL miR mimic or control cel-miR mimic and subjected the posttransfection RNA to a comprehensive 800 miR expression profiling (NanoString). A scatter plot shows miRs with altered digital counts of more than 2.0-fold in cells expressing MetS-FL miR mimics versus counts in control cells expressing the cel-miR mimic (see Figures 1(a)–1(c)). Two hundred and eighty-eight miRs in cells expressing MetS-FL miR mimics versus cells expressing cel-miR mimic, with count ratios ≥ 2.0 or ≤ -2.0, are listed in Supplementary Table S1. The three MetS-FL miRs were also found to significantly affect each other's expression levels (see Supplementary Table S2). The 288 miRs were subjected to Venn analysis, which revealed eleven commonly targeted miRs by the three MetS-FL miR mimics (see Figure 1(d) and Table 1). These eleven miRs have known functional roles in metabolic and cytoskeletal pathways (see Table 2).

### 3.2. MetS-FL miR Mimics Induce Barrier Changes and Modified Structural Appearance of Cell Junction, ZO-1, and Occludin

**Figure 2:**
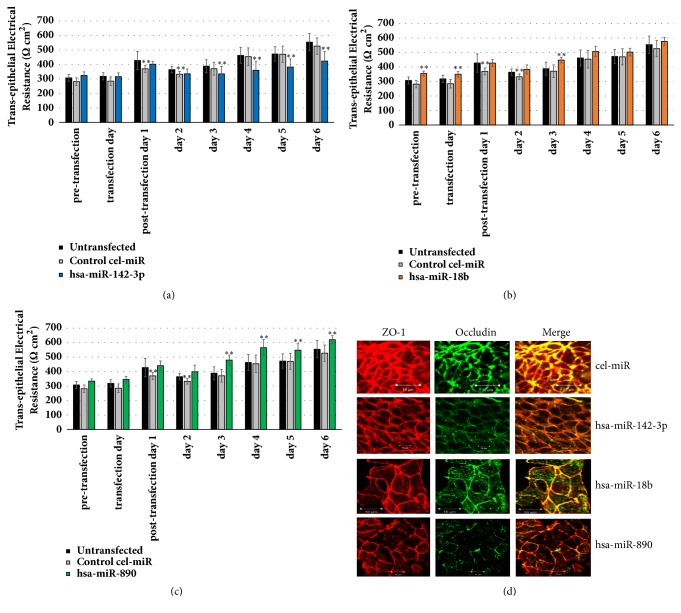
Transfection of MetS-FL miR mimics into Caco-2 colon epithelial monolayers leads to miR-specific changes in barrier integrity and epithelial cell junction. (a, b, c) Transepithelial electrical resistance (TEER) was measured daily pre- and posttransfection of cells with control miR mimic or MetS-FL miR mimics. Background TEER measurements in “blank” wells without cells were subtracted from TEER values in transfected cells. The subtracted TEER values are normalized against seeding surface area and expressed as Ohm-cm^2^. Data are means ± standard deviations calculated from at least four biological replicates. Bar graphs show TEER values of untransfected cells and cells transfected with control* C.elegans* cel-miR-67 mimic or each of the MetS-FL miR mimics. *∗∗* indicates significant (p <0.05) changes in barrier TEER in miR mimic-transfected compared to nontransfected cells. (d) Transfection of MetS-FL miR mimics into Caco-2 colon epithelial monolayers leads to altered appearance of Zona Occludens-1 (ZO-1) and Occludin within the cell junction. Images show immunofluorescence staining and confocal microscopy visualization of ZO-1 (red), Occludin (green), or both (yellow). Scale bars are 10 *μ*m.

Global miRNome changes exerted by MetS-FL miR mimics include eleven target miRs with known metabolic and cytoskeletal signaling roles. To further investigate epithelial and cell junction effects exerted by the MetS-FL miR trio, Caco-2 cells were grown as polarized monolayers on a filter membrane. Apico-basolateral polarity across Caco-2 monolayers was examined by measuring TEER [30] daily. After a 7-day period of TEER stabilization, the polarized cells were transfected with individual MetS-FL miR mimic or control cel-miR mimic. Following cell transfection, changes in barrier integrity were captured by monitoring TEER daily for 6 days. Our data show that transient transfection of cells with each of the MetS-FL miR trio mimics leads to variable changes in TEER (see Figures 2(a)–2(c)). Notably, transfection of cells with hsa-miR-142-3p mimic led to a significant 19-29% reduction in TEER (see Figure 2(a)).

TEER values reflect epithelial cohesion and polarity, which are maintained by structural components within the epithelial cell junction [10, 30]. We investigated the cell junction architecture by staining some of the structural components, ZO-1 and Occludin, both of which had been previously associated with NAFLD patients [24] or animal models [23]. Following transfection of cells with each MetS-FL miR mimic, we found evidence of disruption of the continuous junctional appearance of both proteins (see Figure 2(d)). In particular, following transfection with hsa-miR-142-3p and hsa-miR-890, Occludin staining was absent along the cell junction locations where ZO-1 remained intact (see Figure 2(d)).

### 3.3. ZO-1 and Occludin mRNAs Are Not Significantly Altered in Cells Expressing MetS-FL miRs

**Figure 3:**
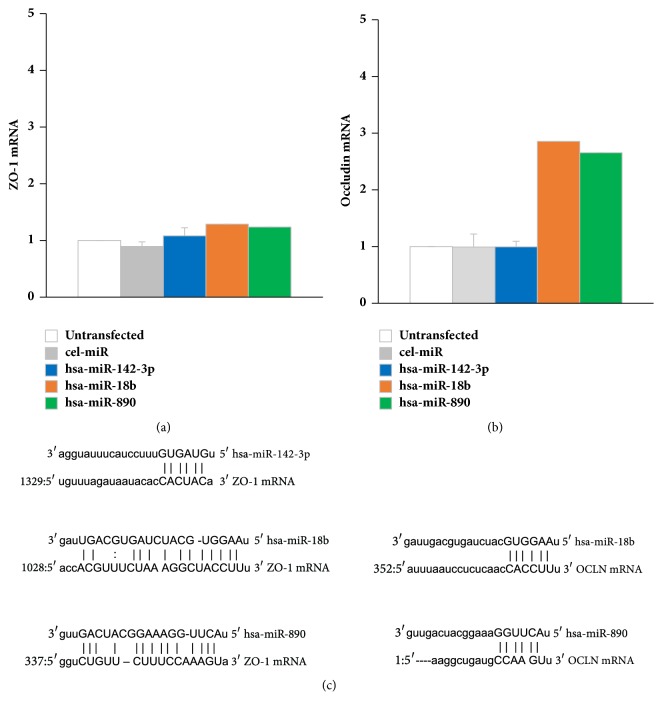
Transfection of MetS-FL miR mimics into CRL-1790 cells did not directly affect cellular ZO-1 (a) or Occludin (b) mRNA levels, although mRNA target sequence alignments with each of MetS-FL miRs were predicted (c).

We investigated whether any of the MetS-FL miRs had direct effects on the expression levels of ZO-1 mRNA or Occludin (OCLN) mRNA, with expression levels of housekeeping gene GAPDH used as a control. Although target sequence alignments were predicted by a database (microrna.org; see Figure 3(c)), neither the endogenous ZO-1 nor Occludin mRNA levels were significantly altered by transient transfection of CRL-1790 cells with any of the MetS-FL miRs (see Figures 3(a) and 3(b)). Two of the miRs (miR-18b, miR-890) did exhibit a 2-3-fold increase in Occludin mRNA expression (see Figure 3(b)). While degradation of target transcripts is often the result of miR targeting [35, 36], cases of miR-mediated gene upregulation have also been identified [37]. A possible miR-mediated suppression of an Occludin transcriptional activator cannot be ruled out. We conclude that in normal colorectal cells, there is no observable direct effect of MetS-FL miRs on ZO-1 or Occludin cellular transcripts.

### 3.4. Signaling Networks and Canonical Pathways Are Predicted by hsa-miR-142-3p, hsa-miR-18b, hsa-miR-890, Eleven Common miR Targets, ZO1, and Occludin

**Figure 4:**
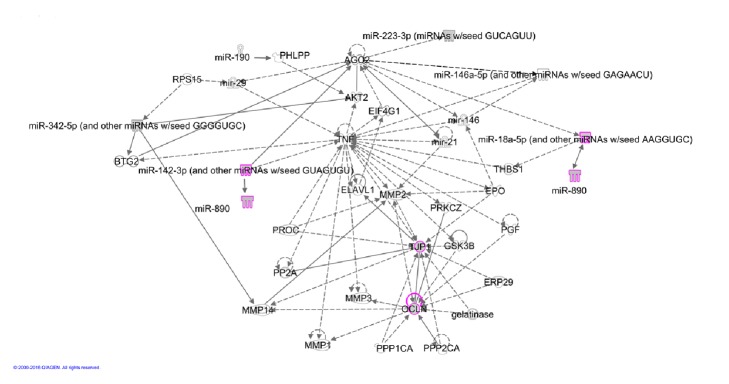
A signaling network was constructed by IPA core analysis from the MetS-FL miR trio, the eleven common miR targets of MetS-FL miRs, ZO1, and OCLN. Using Pathway Designer (IPA), miR-890 and miR-190 were added to the network based on present data (Figure 1) and literature [32].

To elucidate the potential signaling network affecting colorectal epithelial cells and the intestinal barrier based on the MetS-FL miR trio signature, we generated a network representation using QIAGEN's Ingenuity Pathway Analysis (IPA®, QIAGEN Redwood City, www.qiagen.com/ingenuity). IPA is a network database that integrates a broad variety of data from public databases and aggregates the data into network representations based on input nodes (usually genes). Data for the network architecture may be derived from protein-protein interactions, transcriptional activation or repression, or other regulatory interactions from human and other mammalian model systems. An integrated pathway analysis (IPA) was performed using the eleven common target miRs (see Table 1), MetS-FL miR trio, ZO-1, and Occludin. Pathway analysis revealed a signaling network centered on AKT2 and TNF (see Figure 4). The top canonical pathways reveal signaling related to cell-cell junction signaling, inflammation, injury, and hyperplasia. Using Pathway Designer (IPA), the hsa-miR-890 was added to the pathway based on global miRNome profiling presented in Figure 1(c). The hsa-miR-190, one of the eleven common miR targets, was also added to the pathway based on a recent literature report of its direct involvement in AKT signaling and inflammation [32].

## 4. Discussion

The present work demonstrates potential functional implications of our previously reported MetS-FL trio miR signature (hsa-miR-142-3p, hsa-miR-18b, and hsa-miR-890) [13, 25] upon colorectal epithelial cells and intestinal barrier function. Transient expression of each of the three miRs led to variable modifications of apico-basolateral polarity, which affects barrier cohesion and integrity. Although all miR expressions in the polarized epithelial model led to intermittent disappearance of ZO-1 and Occludin along the cell junction, none of the miRs had direct effects on Occludin or ZO1 mRNA levels. A global miRNome network that potentially exerts indirect effects on observed barrier changes was revealed, including a total of 288 miRs. Expression levels of the 288 miRs were altered by more than 2-fold in the human colon epithelial cells expressing the MetS-FL miR mimics, compared with the control cel-miR mimic. The MetS-FL miR mimics also affected each other's endogenous colorectal cell expressions. Of the 288 miRs, eleven miRs with known metabolic and cytoskeletal roles were found to be common targets of all three MetS-FL miRs. An epithelial-focused signaling network was assembled from the MetS-FL miR trio, the eleven common miR targets, ZO-1, and Occludin, which revealed cell junction signaling, inflammation, injury, and hyperplasia as major canonical pathways. In summary, our work suggests a MetS-FL miR signature to be associated with a functional epithelial cell junction and colorectal-specific signaling network.

Our network analysis revealed AKT and TNF*α* as mediators of this signaling pathway. TNF*α* is central to inflammation which mediates liver injury in the two-hit hypothesis proposed for NAFLD [38]. TNF*α* expression is enhanced under inflammasome deficiency due to activation of the TLR signaling pathways by microbial products, and this leads to exacerbation of liver steatosis and steatohepatitis [39]. In a Caco-2 monolayer model, TNF*α* has been shown to increase tight junction permeability and decrease TEER [40]. Such demonstrated roles in barrier maintenance in the context of the gut-liver axis have been the basis for anti-TNF therapy such as infliximab, which is effective in reversing liver steatosis and correcting insulin resistance in animal models [41]. More recently, TNF was shown as a mediator for the combinatorial effects of endoplasmic reticulum (ER) stress and hypernutrition in liver hyperplasia and hepatocellular carcinoma [42]. Whether injury or inflammation occurs prior to, or as a result of, altered barrier permeability is unclear; however, a clear role for both TNF and AKT in the unfolded protein response (UPR) and ER stress response is increasingly recognized [43]. The UPR and ER stress response are evolutionarily conserved cellular adaptations which balance survival versus death in response to stress, such as nutrient stress [43]. The critical role of AKT in the ER stress response is demonstrated by its ability to act both as an activator of metabolic pathways to increase de novo lipogenesis and glycolysis [44] and as an effector of nutrient signals such as metabolic stress induced by low glucose in the cell environment [45].

We have previously reported AKT as a predicted target in a signaling pathway constructed by peripheral miRs found in cohorts of patients with chronic gastrointestinal dysfunction and altered intestinal permeability [26, 46]. Of note, one of these peripheral miRs, miR-342, is one of the eleven common targets of MetS-FL miR trio in the present patient cohort. In addition to AKT and TNF, the present network also reveals MMP2 and hsa-miR-155, which we have previously reported to be altered in NAFLD (Longchamps 2012, Longchamps 2014) [13, 47]. Both Occludin and ZO-1, the two key protein components of the tight junction, are downstream to TNF signaling, with Matrix Metallo-Proteinases (MMP2, MMP1, and MMP3) appearing as intermediates. Thus, our network provides a generalized context for the hypothesis that dysregulation of miRs may result in alteration of epithelial permeability via effects of TNF and AKT signaling.

Given that the MetS-FL miR trio and their common targets implicate TNF, AKT, injury, and hyperplasia as top canonical pathways and functions, our findings have implications for utilizing the miR trio signature as noninvasive biomarkers (see Table 3). First, the MetS-FL miR trio presents values for colon-specific context considerations when testing therapy directed against molecules such as TNF and AKT. Particularly for oral therapy, the demonstrated function on colon barrier modulation warrants considerations of its utilization as a biomarker before and during therapy. Second, current interest is rising on using miRs as targeted therapy. Anti-miR-155 has been suggested for hepatocellular carcinoma and alcoholic fatty liver disease [48, 49]. Our results lend support to miRNome network analysis, especially if a therapy is targeted at the colon. The current miR signature and network may also be considered in nutrient-derived molecule analysis such as resveratrol, which is suggested to promote intestinal health. While our current study is limited in sample size, the functional relevance and miR network signature could be incorporated in studies examining the role of intestinal health promoting nutrients such as resveratrol, a component of red wine and grape skin [50].

## 5. Conclusions

**Figure 5:**
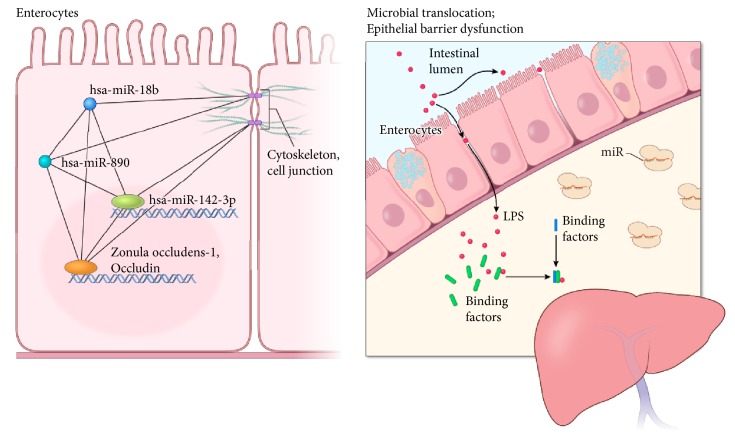
Working model of the MetS-FL miRs in colorectal epithelia. On left, signaling network in which the three miRs participate shows a global cellular miRNome network targeting the epithelial cell junction in colon enterocytes. On right, microbial translocation and epithelial barrier dysfunction are associated with the MetS-FL miR network. LPS: lipopolysaccharides. (Acknowledgment: A. Hoofring, NIH Medical Arts Branch).

Based on the findings discussed, a working model for the MetS-FL miRs in the context of the gut barrier-liver axis is presented (see Figure 5). Changes in colon epithelial barrier structure from local and concerted effects of circulating MetS-FL miRs may be associated with increased intestinal permeability and microbial translocation. Serum LPS Binding Protein or LBP [51] has been shown by others [52–54] to be elevated as a correlate of microbial translocation and inflammatory response, which may be a signal of ongoing metabolic stress or injury in the context of MetS and NAFLD. Overall, the present work supports the importance of miRs at the molecular level in MetS and fatty liver, and the* in vitro* data provides mechanistic support for the proposed gut-liver communication in NAFLD pathogenesis. Future work shall expand the present study in other models.

## Figures and Tables

**Table 1:** Eleven miRs commonly altered in expression levels (≥ 2.0-fold) in CRL-1790 cells transfected with MetS-FL miR mimics (miR-142-3p, miR-18b, and miR-890) compared with cells expressing control cel-miR mimic.

miR Name	miR-142-3p vs. cel-miR	P value of: miR-142-3p vs. cel-miR	miR-18b vs. cel-miR	P value of: miR-18b vs. cel-miR	miR-890 vs. cel-miR	P value of: miR-890 vs. cel-miR
hsa-miR-10b-5p	3.61	0.051	3.26	0.0736	4.06	0.0427
hsa-miR-1254	-3.8	0.0341	-2.55	0.0906	-1.96	0.3425
hsa-miR-1301-3p	2.14	0.3616	2.83	0.3184	3	0.1013
hsa-miR-18b-5p	-2.81	0.6243	2088.21	0.0454	-3.69	0.5328
hsa-miR-190a-3p	2.03	0.3964	2.16	0.3888	1.99	0.4261
hsa-miR-223-3p	2.9	0.05704	4.02	0.0266	2.03	0.1795
hsa-miR-342-5p	2.52	0.2326	3.12	0.2596	3.85	0.0781
hsa-miR-3614-3p	2.01	0.2026	2.35	0.0953	3.09	0.0476
hsa-miR-566	2.74	0.1073	2.38	0.3412	4.06	0.0151
hsa-miR-675-5p	5.41	0.022	2.79	0.0764	2.68	0.2566
hsa-miR-890	3.87	0.1851	3.57	0.386	15456.06	0.0031

^*∗*^ *P*-values are calculated from 3 independent (performed on separate dates) replicate transfection experiments.

**Table 2:** Metabolic and cytoskeletal signaling roles of eleven common miR targets of the MetS-FL miR trio.

	Metabolic Role	Cytoskeletal or Cell Junction Role
hsa-miR-10b-5p	Striatal metabolism in Huntington's Disease [35]	Regulates cytoskeletal RhoC in glioblastoma [36]

hsa-miR-1254	Interacts with structured elements in cell cycle and apoptosis regulator 1 (CCAR1) 5' Untranslated Region [37]	Associated with miR export and processing between nuclear and cytoplasmic compartments [32]; Associated with invasive Retinoblastoma pathogenesis [38]

hsa-miR-1301-3p	Altered level in Low-Onset Hypogonadism [39]; Downregulates maternal circulating leptin levels [40]	Inhibits migration and invasion of HepG2 hepatic carcinoma cells [41]

hsa-miR-18b-5p	Interacts with insulin growth factor-1 to downregulate proliferation of human retinal endothelial cells grown in high-glucose media [12]; Increases plaque buildup, leading to atherosclerosis [42]	Modulates genes involved in cell migration and upregulated in breast cancer [43]

hsa-miR-190a-3p	Under regulation of estrogen receptor (ER) signaling [44]	Inhibits epithelial-mesenchymal transition of hepatoma cells [45]; Targets protease-activated-receptor 1 (PAR-1), which is a metastasis promoting protein [44]

hsa-miR-223-3p	Inversely correlated with insulin-like growth factor 1 levels [46]; Regulates osteoclast and osteoblast differentiation, leading to regulation of bone metabolism [47]; Downregulates glucose uptake controlled by insulin in the adipose tissue of insulin-resistant women [48]; Upregulates glucose uptake in cardiomyocytes via the glucose transporter 4 protein [49]; Regulates multiple pathways involved in cholesterol uptake and metabolism [50]; Downregulates high-density lipoprotein uptake [51]	Regulates epithelial mesenchymal transition [52]

hsa-miR-342-5p	Involved in regulatory T cell function in Type I Diabetes Mellitus [53]	Targets DNA methyltransferase 1 and inhibits colorectal cancer cell proliferation and invasion [54]

hsa-miR-3614-3p	Regulates the *OLR1* gene which encodes for the lectin-like oxidized low-density lipoprotein receptor-1 protein [55]	Involved in c-Myc-regulated migration and angiogenesis [56]

hsa-miR-566	Activates epidermal growth factor receptor signaling in glioblastoma cells [57]	Associated with metastatic colon cancer patient profile [58]

hsa-miR-675-5p	Inhibits the expression of insulin-like growth factor 1 receptor and insulin receptor in rhabdomyosarcoma cells [59]	Upregulates collagen proteins in the cartilage matrix of human articular chondrocytes [60]; Regulates tight junction proteins in intestinal epithelial barrier [61]

hsa-miR-890	Regulates tumor DNA repair [62]	Regulates autophagy and metastasis-associated Rad23B protein in prostate cancer [62]

**Table 3:** Top canonical pathways, top diseases and biological functions, and top toxicology functions revealed by the eleven common miR targets of the MetS-FL trio, ZO-1, and Occludin.

**Top Canonical Pathways**	p-value
Tight Junction Signaling	0.00255
Sertoli Cell-Sertoli Cell Junction Signaling	0.00277
Germ Cell-Sertoli Cell Junction Signaling	0.0763

**Top Upstream Regulators**	p-value

Gelatinase	5E-07
ERP29^*∗*^	4.66E-06
MMP9^*∗*^	0.000013
CAV1^*∗*^	1.75E-05
MMP2^*∗*^	2.54E-05

**Top Diseases and Disorders**	p-value

Inflammatory Response	0.00000116 - 0.0013
Cancer	0.00000659 - 0.0474
Hematological Disease	0.00000659 - 0.0316
Immunological Disease	0.00000659 - 0.0426
Organismal Injury and Abnormalities	0.00000659 - 0.0472

**Top Molecular and Cellular Functions**	p-value

Cell Death and Survival	0.000000194 - 0.0121
Cell-To-Cell Signaling and Interaction	0.00000116 - 0.0318
Cellular Function and Maintenance	0.00000116 - 0.0300
Energy Production	0.000190 - 0.000190
Molecular Transport	0.000190 - 0.000190

**Top Tox Functions (Hepatotoxicity)**	p-value

Liver Necrosis/Cell Death	0.00420 – 0.00420
Hepatocellular Carcinoma	0.345 – 0.345
Liver Hyperplasia/Hyperproliferation	0.345 – 0.596

^*∗*^ERP29: endoplasmic reticulum protein 29; MMP: matrix metallo-proteinase; CAV1: caveolin-1.

## Data Availability

The NanoString data used to support the findings of this study are included within the article. The transepithelial electrical resistance data and quantitative real-time polymerase chain reaction (qPCR) data used to support the findings of this study are available for public use per the National Institutes of Health Data Sharing Policy by contacting the corresponding author (hendersw@mail.nih.gov).

## Supplementary Materials

Supplementary Materials Supplementary Table 1: Two hundred and eighty-eight miRs are altered in their expression levels by ≥ 2.0-fold in CRL-1790 cells expressing any of the MetS-FL miR (miR-142-3p, miR-18b, and miR-890) mimics compared to cells expressing control cel-miR mimic. Supplementary Table 2: effect of MetS-FL miRs on each other's endogenous expression levels in CRL-1790 cells.
