# Supplementary material for: Colon Epithelial MicroRNA Network in Fatty Liver
Source: Can J Gastroenterol Hepatol. 2018 Sep 24;2018:8246103. doi: 10.1155/2018/8246103 (PMC6174781; doi:10.1155/2018/8246103)
Supplement: Supplementary Materials — Supplementary Table 1: Two hundred and eighty-eight miRs are altered in their expression levels by ≥ 2.0-fold in CRL-1790 cells expressing any of the MetS-FL miR (miR-142-3p, miR-18b, and miR-890) mimics compared to cells expressing control cel-miR mimic. Supplementary Table 2: effect of MetS-FL miRs on each other's endogenous expression levels in CRL-1790 cells. [file 8246103.f1.pdf]

SUPPLEMENTARY MATERIALS

Supplementary Table 1: Two hundred and eighty-eight miRs are altered in their expression levels by ≥ 2.0-fold in CRL-1790 cells expressing any of the MetS-FL miR (miR-142-3p, miR-18b, and miR-890) mimic compared to cells expressing control cel-miR mimic.

miR Name	miR-142-3p vs. cel-miR	P value of: miR-142-3p vs. cel-miR	miR-18b vs. cel-miR	P value of: miR-18b vs. cel-miR	miR-890 vs. cel-miR	P value of: miR-890 vs. cel-miR
hsa-let-7c-5p	2.19	0.03606	1.02	0.957506	-1.17	0.666809
hsa-miR-1-3p	1.09	0.802673	2.16	0.314305	1.37	0.341617
hsa-miR-101-3p	1.84	0.084051	-2.41	0.375395	1.05	0.837828
hsa-miR-105-5p	-2.14	0.175302	1.17	0.773273	-1.58	0.351355
hsa-miR-10b-5p	3.61	0.050967	3.26	0.073587	4.06	0.042708
hsa-miR-1178-3p	-1.65	0.457899	-1.25	0.817197	-2.23	0.241221
hsa-miR-1185-5p	2.27	0.061002	3.7	0.014811	1.62	0.108062
hsa-miR-1193	22.65	0.023702	2.34	0.321815	1.28	0.701636
hsa-miR-1200	-1.23	0.800336	4.27	0.064434	-1.08	0.874252
hsa-miR-1202	2.1	0.26361	1.24	0.682648	1.02	0.965436
hsa-miR-1203	2.07	0.227216	1.96	0.277651	1.7	0.364619
hsa-miR-1204	1.11	0.856621	1.38	0.535461	2.41	0.138737
hsa-miR-1224-3p	-1.97	0.278859	2.66	0.158544	-1.16	0.805958
hsa-miR-1234-3p	1.9	0.082749	2.47	0.03302	1.28	0.420733
hsa-miR-1236-3p	1.22	0.789551	3.63	0.123661	1.74	0.433375
hsa-miR-1245a	1.13	0.886945	2.66	0.320127	1.42	0.69794
hsa-miR-1245b-3p	1.54	0.608792	2.05	0.137482	2.62	0.051956
hsa-miR-1246	-2.14	0.300002	1.1	0.90355	-1.27	0.752287
hsa-miR-1249-3p	-1.51	0.250381	1.99	0.037103	-1.21	0.68247
hsa-miR-1250-5p	1.39	0.571302	-1	0.994735	2.63	0.339097
hsa-miR-1254	-3.8	0.034118	-2.55	0.090602	-1.96	0.342467
hsa-miR-125a-3p	4.61	0.01646	-1.15	0.766352	-1.05	0.902663
hsa-miR-1262	-2.23	0.145918	1.06	0.91548	-1.43	0.467416
hsa-miR-1268b	-1.97	0.362082	1.41	0.337539	-1.77	0.222333
hsa-miR-1269b	1.7	0.560032	3.63	0.072062	3.76	0.009439
hsa-miR-127-5p	-2.08	0.310629	1.14	0.784063	-1.35	0.534999
hsa-miR-1275	1.25	0.777891	3.63	0.038421	1.12	0.803694
hsa-miR-1276	1.47	0.317249	-1.01	0.984591	5.14	0.004201
hsa-miR-1277-3p	1.87	0.343163	2.35	0.191317	1.1	0.869095
hsa-miR-1278	-1.13	0.855618	1.44	0.620098	-2.6	0.201917
hsa-miR-128-1-5p	1.35	0.572872	1.33	0.635153	2.05	0.188209
hsa-miR-128-3p	-3.04	0.33573	-1.81	0.605729	4.05	0.357891
hsa-miR-1281	1.8	0.299035	4.44	0.100427	2.75	0.250801
hsa-miR-1283	-3.6	0.497916	-1.03	0.990534	-3.9	0.473793

hsa-miR-1285-3p	-1.3	0.751771	1.73	0.418979	2.45	0.08705
hsa-miR-1288-3p	1.38	0.707768	1.48	0.473416	2.96	0.030991
hsa-miR-1293	1.43	0.462511	-2.88	0.202524	-2.12	0.206983
hsa-miR-1296-5p	-1.07	0.923674	2.01	0.474003	-1.08	0.910611
hsa-miR-1297	-1.28	0.812216	-2.48	0.407822	-1.62	0.735169
hsa-miR-1298-5p	1.22	0.698921	2.07	0.207884	1.29	0.279199
hsa-miR-1301-3p	2.14	0.36156	2.83	0.318387	3	0.101324
hsa-miR-1302	-2.38	0.351722	-1.02	0.943719	-1.15	0.593141
hsa-miR-1303	1.52	0.480424	1.48	0.415226	5.06	0.087203
hsa-miR-1304-5p	5.6	0.010992	-2.3	0.257813	-1.22	0.549384
hsa-miR-132-3p	3.14	0.09392	-1.01	0.98626	-1.2	0.764064
hsa-miR-133b	-1.98	0.278937	-1	0.999387	1.17	0.747523
hsa-miR-134-5p+hsa-miR-6728-5p	-2.16	0.106277	-1.17	0.603536	-1.78	0.013976
hsa-miR-137	-1.6	0.397641	2.13	0.102161	-1.36	0.658952
hsa-miR-142-3p	8821.28	1.41E-05	2.38	0.083829	-1.31	0.414854
hsa-miR-142-5p	-2.92	0.193261	-1.41	0.48133	1.09	0.77702
hsa-miR-1469	1.68	0.432735	1.42	0.634753	6.16	0.013479
hsa-miR-146b-3p	2.88	0.147035	-1.43	0.697336	-1.19	0.651497
hsa-miR-147a	1.28	0.807198	2.04	0.392805	1.19	0.831389
hsa-miR-147b	-1.12	0.669977	2.04	0.154425	-1.13	0.629213
hsa-miR-150-5p	-1.07	0.935937	1.5	0.716819	2.5	0.261464
hsa-miR-152-5p	-1.5	0.55627	1.56	0.543005	2.12	0.027082
hsa-miR-1537-3p	1.35	0.470971	1.1	0.852111	-2.36	0.093229
hsa-miR-155-5p	1.11	0.834609	1.35	0.687076	2.36	0.410397
hsa-miR-181b-2-3p	2.1	0.372367	1.5	0.604057	1.64	0.528189
hsa-miR-181d-3p	1.59	0.498755	2.08	0.430112	1.39	0.48011
hsa-miR-182-3p	1.18	0.796372	2.51	0.232503	-1.21	0.80764
hsa-miR-184	-1.82	0.459115	-1.04	0.931146	2.62	0.458814
hsa-miR-187-3p	1.13	0.77966	2.95	0.05577	1.11	0.898863
hsa-miR-188-3p	-1.32	0.636893	8.62	0.026798	1.26	0.637197
hsa-miR-18a-5p	-1.12	0.762193	18.82	0.094492	-1.14	0.728732
hsa-miR-18b-5p	-2.81	0.624342	2088.21	0.045375	-3.69	0.532792
hsa-miR-1908-3p	1.79	0.432721	1.51	0.706337	7.25	0.032135
hsa-miR-1909-3p	-1.66	0.406892	1.12	0.893527	-2.13	0.206412
hsa-miR-190a-3p	2.03	0.396382	2.16	0.388796	1.99	0.426103
hsa-miR-190b	2.59	0.149323	4.47	0.047128	1.81	0.409188
hsa-miR-1915-3p	1.08	0.786043	1.46	0.276024	-2.18	0.298938
hsa-miR-193a-3p	-1.65	0.178676	-1.34	0.610954	-2.77	0.223047
hsa-miR-194-5p	1.38	0.468615	2.05	0.038472	36.82	0.001269
hsa-miR-195-5p	1.51	0.631861	1.09	0.915847	2.17	0.382363
hsa-miR-196a-3p	1.52	0.650474	1.98	0.612906	1.64	0.57162
hsa-miR-1973	-1.34	0.277637	-1.01	0.985636	-2.6	0.146431

hsa-miR-1976	1.43	0.554708	3.59	0.081194	1.73	0.37972
hsa-miR-200b-3p	-1.07	0.773546	8.53	0.002443	-1.3	0.375684
hsa-miR-202-3p	-1.34	0.670352	-2.44	0.260239	2.34	0.201064
hsa-miR-204-5p	-1.78	0.435923	-2.03	0.362011	-1.8	0.171815
hsa-miR-208a-3p	-2.09	0.051457	-1.21	0.620922	-1.56	0.192948
hsa-miR-208b-3p	-2.26	0.299544	1.05	0.9236	-2.6	0.093203
hsa-miR-208b-5p	1.02	0.982457	1.6	0.448545	2.03	0.247614
hsa-miR-21-5p	1.03	0.966862	-1.02	0.976063	2.94	0.206103
hsa-miR-2113	-1.01	0.982946	6.18	0.000219	1.36	0.352738
hsa-miR-217	4.24	0.173285	-1.14	0.864819	-1.9	0.480815
hsa-miR-218-5p	1.24	0.556827	1.56	0.300269	15.54	0.002212
hsa-miR-219a-1-3p	1.2	0.738957	1.41	0.302833	2.88	0.027576
hsa-miR-223-3p	2.9	0.057042	4.02	0.026566	2.03	0.17953
hsa-miR-224-5p	1.13	0.885581	2.04	0.350934	1.2	0.790719
hsa-miR-296-3p	-2.55	0.065092	-1.59	0.644263	-1.43	0.423712
hsa-miR-298	2.66	0.283879	3.15	0.070277	-1.15	0.776573
hsa-miR-300	1.45	0.130441	1.24	0.478924	8.62	0.183916
hsa-miR-301a-5p	2.19	0.082789	1.72	0.176924	2	0.13704
hsa-miR-301b-3p	1.05	0.927397	1.95	0.184928	-1.15	0.772476
hsa-miR-301b-5p	-1.42	0.606466	1.63	0.117482	-2.51	0.086843
hsa-miR-302b-3p	-1.64	0.684474	-2.61	0.406858	-1.19	0.889393
hsa-miR-30a-5p	-1.95	0.017961	-1.3	0.526955	-1.98	0.007032
hsa-miR-30e-3p	2.3	0.08615	2.33	0.108256	1.38	0.4159
hsa-miR-31-5p	1.01	0.972319	-3.02	0.061301	1.74	0.009215
hsa-miR-3131	1.76	0.355629	1.42	0.62432	2.74	0.03507
hsa-miR-3136-5p	1.34	0.773071	2.35	0.352065	2.09	0.38519
hsa-miR-3140-3p	-1.18	0.683728	1.45	0.598495	2.51	0.03865
hsa-miR-3150b-3p	-1.66	0.434147	1.62	0.383194	6.37	0.023245
hsa-miR-3164	-1.24	0.797679	1.13	0.884056	3.15	0.223938
hsa-miR-3179	1.73	0.1914	3.31	0.128382	2.88	0.038979
hsa-miR-3180-5p	2.4	0.202053	2.77	0.156818	1.92	0.314295
hsa-miR-3185	-1.34	0.719902	2.26	0.387619	1.15	0.866245
hsa-miR-3202	2.14	0.094806	1.56	0.260562	9.09	0.007245
hsa-miR-320b	1.22	0.726146	1.42	0.544768	2.52	0.050253
hsa-miR-320d	1.43	0.44431	2.01	0.066472	1.96	0.216809
hsa-miR-323a-5p	-2.25	0.181724	-1.4	0.474068	-1.33	0.525161
hsa-miR-323b-5p	-2.09	0.441542	-1.05	0.913903	-1.65	0.509427
hsa-miR-324-3p	-1.98	0.43631	-1.48	0.427099	-1.32	0.514894
hsa-miR-328-3p	-1.05	0.903986	1.36	0.541172	4.27	0.010402
hsa-miR-328-5p	1.39	0.68456	1.95	0.417771	1.07	0.944542
hsa-miR-329-3p	-1.7	0.518872	-1.07	0.922975	12.01	0.121209
hsa-miR-329-5p	2.39	0.130342	2.44	0.161713	-1.54	0.378869
hsa-miR-330-3p	-1.45	0.60302	1.29	0.684974	-2.44	0.041458

hsa-miR-338-5p	1.05	0.920275	2.21	0.288661	-1.26	0.754942
hsa-miR-339-5p	2.35	0.203391	2.03	0.261774	1.3	0.663327
hsa-miR-33a-5p	-1.05	0.910402	2.05	0.182962	1.76	0.266073
hsa-miR-342-5p	2.52	0.232602	3.12	0.2596	3.85	0.078097
hsa-miR-346	1.65	0.32791	1.91	0.414193	2.64	0.12783
hsa-miR-34b-3p	2.24	0.047764	1.17	0.834105	-1.49	0.554591
hsa-miR-34c-3p	1.41	0.252045	1	0.994946	7.31	0.035988
hsa-miR-3613-3p	-1.42	0.354328	2.2	0.142368	-1.08	0.800846
hsa-miR-3613-5p	1.04	0.945062	2.31	0.23923	1.3	0.636631
hsa-miR-3614-3p	2.01	0.202605	2.35	0.095252	3.09	0.047567
hsa-miR-3614-5p	1.73	0.343415	2.05	0.146506	1.31	0.477863
hsa-miR-363-5p	-1.39	0.29677	-1.34	0.547448	4.41	0.009044
hsa-miR-369-3p	1.35	0.570043	1.87	0.302834	2.08	0.230918
hsa-miR-3690	-1.9	0.114263	1.11	0.855424	3.04	0.011642
hsa-miR-371b-5p	1.44	0.475679	-1.13	0.852601	11.67	0.167168
hsa-miR-374c-5p	-3.13	0.016827	-1.54	0.638553	1.49	0.34078
hsa-miR-375	-1.27	0.545392	-2.13	0.144897	-2.69	0.006912
hsa-miR-376c-5p	-1	0.99447	2.35	0.096929	1.13	0.795824
hsa-miR-378c	2.22	0.020034	1.04	0.949078	2	0.159791
hsa-miR-378e	-1.01	0.996657	2.97	0.432893	-3.18	0.384031
hsa-miR-378g	1.55	0.575515	2.2	0.350385	1.43	0.636699
hsa-miR-381-5p	1.3	0.561125	2.03	0.150576	1.64	0.344282
hsa-miR-383-5p	-1.47	0.322492	-1.14	0.847552	2.67	0.06222
hsa-miR-3918	1.45	0.539629	3.37	0.162988	3.24	0.137679
hsa-miR-422a	-2.49	0.578309	1.62	0.750017	-3.81	0.390675
hsa-miR-4284	-2.53	0.223183	-1.41	0.582378	-2.07	0.420702
hsa-miR-433-5p	-1.03	0.954399	-1.64	0.513297	-3.3	0.131691
hsa-miR-4435	-1.55	0.047669	1.1	0.875234	27.06	0.000256
hsa-miR-4448	-1.61	0.207614	1.14	0.571033	-2.05	0.281453
hsa-miR-4458	1.86	0.402299	2.04	0.350334	6.77	0.057594
hsa-miR-4461	-1.21	0.787077	-3.36	0.027881	-1.51	0.303132
hsa-miR-4485-3p	1.11	0.832034	2.48	0.028216	1.13	0.71071
hsa-miR-4488	1.39	0.494631	1.92	0.068505	-2.06	0.06003
hsa-miR-449a	-1.04	0.942072	2	0.276796	1.58	0.406085
hsa-miR-450a-1-3p	1.63	0.433431	2.85	0.235189	5.92	0.052849
hsa-miR-4521	1.11	0.837257	1.27	0.677865	2.12	0.151011
hsa-miR-4524a-5p	1.57	0.304275	4.3	0.029539	2.8	0.068808
hsa-miR-4532	-1.37	0.737126	1.06	0.909275	-2.09	0.043211
hsa-miR-4647	3.65	0.157733	2.82	0.21973	1.67	0.487817
hsa-miR-4707-3p	-1.09	0.880141	-2.18	0.211744	1.63	0.378733
hsa-miR-4792	-1.34	0.685003	4.19	0.125165	-1.42	0.624975
hsa-miR-488-3p	2.11	0.131714	-1.29	0.649305	-2.86	0.02818
hsa-miR-492	1.58	0.363834	1.48	0.300476	13.43	0.001828

hsa-miR-493-3p	-1.15	0.709478	1.08	0.726797	2	0.029821
hsa-miR-494-3p	-5.95	0.505981	-1.6	0.846789	-1.31	0.921342
hsa-miR-499a-3p	-1.45	0.639346	-1.75	0.475234	-2.75	0.391274
hsa-miR-5001-3p	-1.2	0.760117	-3.36	0.12597	1.68	0.513743
hsa-miR-500a-5p+hsa-miR-501-5p	-1.51	0.280899	-1.63	0.582323	3.02	0.047313
hsa-miR-502-3p	1.83	0.211189	2.15	0.266776	1.17	0.72719
hsa-miR-502-5p	-1.01	0.990801	1.64	0.691368	-3.29	0.371644
hsa-miR-504-5p	1.03	0.936063	1.61	0.380067	2.74	0.059961
hsa-miR-508-5p	3.62	0.011386	1.67	0.253217	-1.01	0.957659
hsa-miR-509-3-5p	1.11	0.842119	1.13	0.897864	-2.04	0.254874
hsa-miR-509-3p	2.11	0.106598	-1.67	0.018751	-1.04	0.930179
hsa-miR-510-5p	-1.46	0.563278	2.12	0.341401	-1.19	0.766148
hsa-miR-512-3p	-2.83	0.146622	-1.48	0.382972	-1.78	0.435475
hsa-miR-513a-3p	-1.38	0.501786	2.37	0.269882	1.35	0.758341
hsa-miR-514a-5p	-2.44	0.328102	1.13	0.708341	-1.73	0.205094
hsa-miR-514b-3p	-1.05	0.94539	-1.84	0.342836	-2.44	0.228189
hsa-miR-514b-5p	1.41	0.547591	2.3	0.175212	1.33	0.571189
hsa-miR-515-3p	1.18	0.833984	1.48	0.688159	3.2	0.216493
hsa-miR-515-5p	1.33	0.644454	-1.25	0.809262	3.31	0.072907
hsa-miR-516a-5p	-1.53	0.279043	1.69	0.318815	3.68	0.023906
hsa-miR-516b-5p	1.55	0.487683	2.38	0.12735	103.84	0.096997
hsa-miR-517c-3p+hsa-miR-519a-3p	-2.2	0.341909	-1.28	0.51959	-3.27	0.009775
hsa-miR-518b	-1.32	0.619368	-2.03	0.185541	3.04	0.074033
hsa-miR-518d-3p	-1.27	0.703509	-1.14	0.87077	2	0.27715
hsa-miR-518e-3p	-1.32	0.535138	3.73	0.007129	1.17	0.6326
hsa-miR-518f-3p	4.75	0.114935	1.16	0.31914	-2.04	0.086277
hsa-miR-5196-5p	4.71	0.001894	1.09	0.352609	1.73	0.027402
hsa-miR-519b-5p+hsa-miR-519c-5p+hsa-miR-523-5p+hsa-miR-518e-5p+hsa-miR-522-5p+hsa-miR-519a-5p	-3.1	0.484799	-2.27	0.458059	-1.51	0.714341
hsa-miR-519c-3p	-1.21	0.644319	1.34	0.560773	-2.36	0.096496
hsa-miR-519d-3p	-2.04	0.270705	-1.54	0.575312	-1.03	0.952728
hsa-miR-519e-3p	1.14	0.847901	-1.11	0.903553	3.17	0.142949
hsa-miR-520a-5p	1.87	0.133262	2.47	0.029142	1.45	0.363363
hsa-miR-520b	-1.96	0.339166	-1.65	0.488682	-1.94	0.345669
hsa-miR-520c-3p	-2.54	0.05015	-1.29	0.526684	-1.11	0.797792
hsa-miR-520d-3p	1.34	0.717403	-1.3	0.804824	-1.98	0.440292
hsa-miR-520f-3p	-1.1	0.646804	1.52	0.36405	10.58	0.001285
hsa-miR-520g-3p	1.53	0.440762	1.34	0.638352	-1.16	0.772534

hsa-miR-520h	1.8	0.505084	2.79	0.295598	-1.08	0.92867
hsa-miR-521	3.18	0.117971	8.71	0.023211	1.26	0.666703
hsa-miR-522-3p	-1.95	0.44807	-2.46	0.293702	-1.75	0.577583
hsa-miR-523-3p	-2.31	0.121848	-1.23	0.778888	1.09	0.848908
hsa-miR-524-3p	-2.17	0.225808	-1.23	0.742307	1.82	0.089843
hsa-miR-539-3p	1.2	0.789135	1.16	0.751325	3.1	0.119793
hsa-miR-541-3p	-1.18	0.622987	-2.45	0.123958	1.29	0.485935
hsa-miR-542-5p	2.22	0.019812	1.22	0.626754	-1.55	0.228458
hsa-miR-545-3p	1.79	0.446766	2.2	0.303698	2.74	0.195504
hsa-miR-548a-3p	-2.26	0.344504	-2.39	0.248551	-1.26	0.69821
hsa-miR-548a-5p	-2.3	0.458668	-1.45	0.714466	-2.29	0.538138
hsa-miR-548ah-5p	-1.38	0.463105	-1.48	0.389449	-2.17	0.304997
hsa-miR-548al	2.86	0.122062	1.26	0.789372	1.29	0.733016
hsa-miR-548ar-5p	-1.38	0.806694	-5.4	0.304998	-1.65	0.766502
hsa-miR-548c-5p+hsa-miR-548o-5p+hsa-miR-548am-5p	4.38	0.004591	2.48	0.18417	-1.47	0.522247
hsa-miR-548e-3p	-1.04	0.933797	-1.05	0.965795	4.75	0.052567
hsa-miR-548h-5p	-1.86	0.311733	-1.56	0.501705	-2.02	0.288719
hsa-miR-548j-3p	-1.32	0.866383	-2.03	0.654972	-1.27	0.897526
hsa-miR-548j-5p	1.02	0.967368	-1.19	0.802617	-2.75	0.256805
hsa-miR-548k	-2.33	0.085468	-1.05	0.741269	-1.72	0.461453
hsa-miR-551a	-1.36	0.598883	1.5	0.408311	2.17	0.15631
hsa-miR-551b-3p	2.63	0.033229	1.37	0.362733	-1.54	0.125105
hsa-miR-552-3p	-2.48	0.051055	1.3	0.773529	-1.16	0.758384
hsa-miR-556-3p	1.16	0.74639	-1.08	0.774032	3.29	0.093885
hsa-miR-556-5p	-1.57	0.308996	-1.06	0.886514	-3.35	0.126823
hsa-miR-566	2.74	0.107274	2.38	0.341185	4.06	0.015122
hsa-miR-567	-1.4	0.413008	1.18	0.728675	2.4	0.044058
hsa-miR-570-3p	1.05	0.970531	1.37	0.815304	-2.8	0.22604
hsa-miR-571	2.97	0.097203	1.34	0.599674	-1.17	0.835901
hsa-miR-572	1.27	0.709399	4.33	0.067688	1.28	0.637015
hsa-miR-576-3p	1.35	0.712791	3.01	0.212012	1.59	0.577274
hsa-miR-576-5p	-1.24	0.723611	1.22	0.812563	2.33	0.243954
hsa-miR-577	-1.24	0.720804	1.67	0.460046	-1.95	0.202084
hsa-miR-578	-1.09	0.896761	1.53	0.247748	2.08	0.319806
hsa-miR-579-3p	1.07	0.860363	3.87	0.316652	-1.01	0.965981
hsa-miR-580-3p	-1.01	0.993243	1.42	0.686198	2.15	0.423602
hsa-miR-582-3p	-1.22	0.762599	2.33	0.256554	-1.04	0.951791
hsa-miR-582-5p	1.79	0.480896	2.38	0.298689	1.95	0.416919
hsa-miR-584-5p	-1.19	0.808571	-2.53	0.349164	1.23	0.661392
hsa-miR-592	3.21	0.041893	-1.32	0.479162	5.38	0.105485
hsa-miR-596	1.23	0.769787	1.46	0.643134	2.08	0.230935

hsa-miR-600	1.24	0.710634	2.79	0.157496	1.78	0.361465
hsa-miR-601	-1.1	0.851414	-2.27	0.205371	-1.42	0.511827
hsa-miR-606	-1.43	0.463441	1.64	0.579628	3.97	0.05015
hsa-miR-607	1.05	0.909304	2.09	0.220144	-1.26	0.556967
hsa-miR-608	1.64	0.451863	1.34	0.699515	4.14	0.080839
hsa-miR-615-5p	1.25	0.517212	2.35	0.151687	8.01	0.003056
hsa-miR-617	-1.42	0.498975	1.04	0.935213	2.01	0.196288
hsa-miR-619-3p	-1.11	0.822045	-1.61	0.60655	3.99	0.068477
hsa-miR-625-5p	-1.69	0.394012	-2.25	0.211251	-2.28	0.24158
hsa-miR-626	-1.15	0.717975	2.03	0.150964	1.03	0.918433
hsa-miR-627-5p	-1.58	0.874123	-2.64	0.724687	-1.67	0.86308
hsa-miR-629-5p	1.3	0.63358	2.02	0.371837	2.16	0.209149
hsa-miR-638	1.32	0.586476	1.96	0.313498	-1	0.998841
hsa-miR-640	-1.35	0.606434	-2.01	0.237331	-1.2	0.72009
hsa-miR-642a-3p	-2.12	0.300776	-1.1	0.903601	-1.72	0.42884
hsa-miR-648	1.04	0.938697	1.2	0.750969	3.24	0.077173
hsa-miR-649	-2.01	0.375905	1.47	0.491056	6.2	0.013177
hsa-miR-6503-3p	1.16	0.516123	1.29	0.566953	2.36	0.016734
hsa-miR-6503-5p	1.2	0.760217	2.26	0.248683	1.86	0.432651
hsa-miR-6511a-3p	1.16	0.860193	2.26	0.353437	1.93	0.323761
hsa-miR-654-3p	-1.37	0.692335	1.03	0.951127	5.36	0.132786
hsa-miR-656-3p	-2.1	0.596218	-1.04	0.876303	-1.56	0.076488
hsa-miR-660-5p	-1.97	0.236928	1.02	0.955997	-1.08	0.875001
hsa-miR-663a	1.8	0.135034	2.16	0.287667	2.29	0.067287
hsa-miR-664b-3p	-1.64	0.324358	-2.46	0.47818	-1.83	0.295862
hsa-miR-664b-5p	1.18	0.783399	1.33	0.70252	2.12	0.235403
hsa-miR-671-3p	1.52	0.344373	1.93	0.297291	3.03	0.102312
hsa-miR-6720-3p	-1.1	0.812576	4.34	0.007418	-1.13	0.797645
hsa-miR-675-5p	5.41	0.02201	2.79	0.076411	2.68	0.25661
hsa-miR-764	1.76	0.319766	2.79	0.153748	1.14	0.812939
hsa-miR-765	1.43	0.594742	2.08	0.36742	1.1	0.903661
hsa-miR-766-3p	1.15	0.602568	1.12	0.686959	2.6	0.039628
hsa-miR-767-3p	-1.49	0.576146	-1.84	0.328345	3.43	0.018749
hsa-miR-769-3p	1.39	0.298266	2.75	0.029759	1.1	0.75675
hsa-miR-802	1.79	0.459516	2.44	0.366186	2.43	0.27464
hsa-miR-874-3p	-1.14	0.824334	1.22	0.747043	-2.23	0.45075
hsa-miR-874-5p	2.03	0.107674	1.39	0.541506	1.99	0.204098
hsa-miR-876-3p	1.12	0.810102	-1.01	0.979603	2.96	0.048348
hsa-miR-890	3.87	0.185113	3.57	0.386015	15456.06	0.003142
hsa-miR-892a	-2.48	0.100287	-1.02	0.975613	1.44	0.156415
hsa-miR-9-5p	1.3	0.63854	1.29	0.763421	1.96	0.465561
hsa-miR-922	-1.81	0.095599	2.15	0.232136	-1.9	0.35034
hsa-miR-92a-1-5p	-1.21	0.684783	-2.53	0.157994	-1.52	0.476125

hsa-miR-933	-1.1	0.864532	2.31	0.247692	3.03	0.182817
hsa-miR-939-5p	1.61	0.292182	2.37	0.13067	2.02	0.092317
hsa-miR-940	-1.26	0.544992	1.48	0.612547	4.61	0.004045
hsa-miR-944	-2.07	0.592219	1	0.999685	-1.49	0.691642
hsa-miR-96-5p	2.16	0.282201	1.9	0.29488	2.61	0.161049

*p-values are calculated from 3 independent (performed on separate dates) replicate transfection experiments.

Supplementary Table 2: Effect of MetS-FL miRs on each other's endogenous expression levels in CRL-1790 cells.

	**Fold change of expression by hsa-miR-142-3p mimic**	**Fold change of expression by hsa-miR-18b mimic**	**Fold change of expression by hsa-miR-890 mimic**
hsa-miR-142-3p	8821.28<br>*p = 0.000014	2.38<br>p = 0.0838	-1.31<br>p = 0.4149
hsa-miR-18b	-2.81<br>p = 0.6243	2088.21<br>p = 0.0454	-3.69<br>p = 0.5328
hsa-miR-890	3.87<br>p = 0.1851	3.57<br>p = 0.3860	15456.06<br>p = 0.0031

*p-values are calculated from 3 independent (performed on separate dates) replicate transfection experiments.
